# Choroidal perfusion abnormalities associated with Acute Posterior Multifocal Placoid Pigment Epitheliopathy: a case report

**DOI:** 10.1186/s12886-018-0756-8

**Published:** 2018-04-10

**Authors:** Emilia Maggio, Alessandro Alfano, Antonio Polito, Grazia Pertile

**Affiliations:** Sacrocuore Hospital, Via Don Sempreboni 5 - Negrar, 37024 Verona, Italy

**Keywords:** Acute Posterior Multifocal Placoid Pigment Epitheliopathy, Choroidal perfusion abnormalities, Enhanced Depth Imaging Spectral-Domain Optical Coherence Tomography, Indocyanine Green Angiography, Placoid lesions

## Abstract

**Background:**

Indocyanine Green Angiography (ICG-A) and Enhanced Depth Imaging Spectral-Domain Optical Coherence Tomography (EDI-OCT) are essential imaging techniques for diagnosis, management and understanding of the pathophysiology of many chorioretinal diseases. Herein, we report the ICG-A and EDI-OCT findings from a case of Acute Posterior Multifocal Placoid Pigment Epitheliopathy (APMPPE), in which these imaging techniques enable the visualization of more diagnostic details than those observable with other widely used diagnostic tools.

**Case presentation:**

A 60-year-old white female presented with bilateral blurred vision for few days. Fundus examination showed multiple, yellow-white placoid lesions at the posterior pole of both eyes. The placoid lesions were also evident on Spectral-Domain Optical Coherence Tomography (SD-OCT), Fluorescein Angiography (FA), Fundus Autofluorescence (AF), and ICG-A. A complete ophthalmologic examination was performed and the diagnosis of APMPPE was made based on imaging and clinical features. Notably, all the lesions detected by FA, AF and OCT corresponded to focal areas of hypofluorescence seen on ICG-A, whereas several additional hypofluorescent areas that were not associated with FA, AF or OCT abnormalities, were also detected with ICG-A. On follow-up, the regression of outer retinal abnormalities detected on OCT preceded the restoration of choroidal perfusion abnormalities in the corresponding locations on ICG-A. This long-standing choroidal perfusion defect could not be detected with OCT. EDI-OCT scans revealed characteristic choriocapillaris changes beneath the placoid lesions and an increase in choroidal thickness during the acute phase, with subsequent decrease in the inactive stage of the disease.

**Conclusion:**

Our findings show that ICG-A and EDI-OCT provide detailed morphologic information of choroidal abnormalities in APMPPE and allow accurate evaluation of definitive resolution of the lesions. Moreover, they support the acute choroidal hypoperfusion as the primary mechanism overlying the pathogenesis of the disease, and suggest that the non-perfused areas may extend beyond the damage of the outer retina.

## Background

Enabling visualization of the choroidal circulation, Indocyanine Green Angiography (ICG-A) is an essential imaging technique for diagnosis, management and understanding of the pathophysiology of many chorioretinal diseases. Similarly, Enhanced Depth Imaging Spectral-Domain Optical Coherence Tomography (EDI-OCT) is a useful diagnostic tool to evaluate choroidal thickness changes in various chorioretinal disorders and to study the possible correlation of changes in choroidal structures with the pathogenesis of the diseases.

Acute posterior multifocal placoid pigment epitheliopathy (APMPPE) is a rare disorder of uncertain origin that is characterized by the acute onset of multiple, yellow-white placoid lesions at the level of retinal pigment epithelium (RPE), situated mainly in the posterior retinal pole and rarely anterior to the equator [1]. Although the exact pathological origins remain unknown, previous evidence suggests choroidal perfusion abnormalities as the most likely initial factor in APMPPE, with secondary damage of the RPE resulting in typical placoid lesions [2–6].

Herein, we report the ICG-A and EDI-OCT findings from a case of APMPPE, in which these imaging techniques enabled the visualization of more diagnostic details than those obtained with other widely used diagnostic tools. The findings indicate choroidal hypoperfusion as the primary mechanism overlying the pathogenesis of the disease, and suggest that non-perfused areas may extend beyond the damage of the outer retina.

## Case presentation

**Fig. 1:**
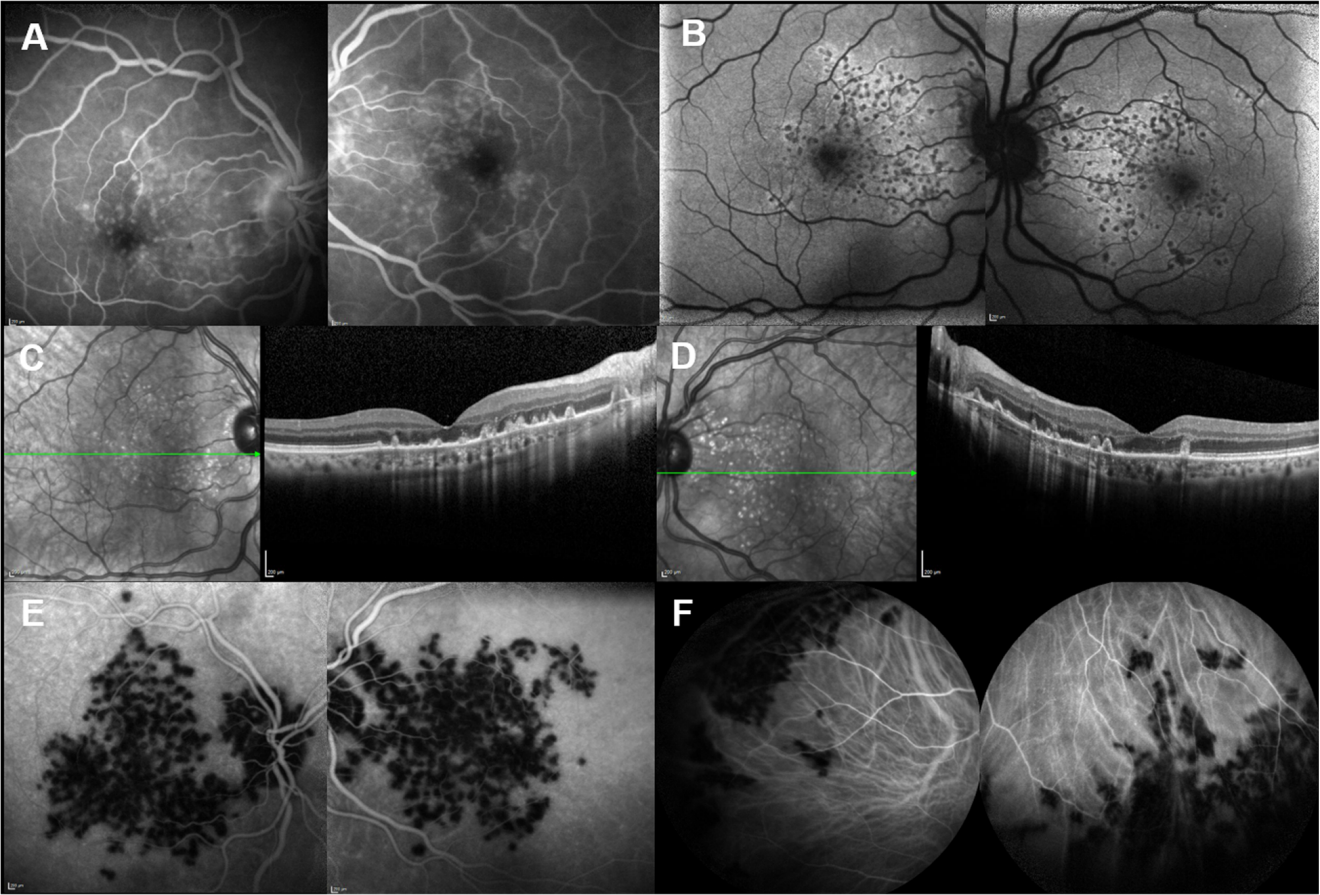
Multimodal imaging findings in the acute phase of disease. **a** Fluorescein Angiography showing late hyperfluorescence of the lesions. **b** Autofluorescence revealing focal round hypoautofluoresces at the posterior pole. **c**-**d** OCT scans showing focal deposits of hyperreflective material at the level of the outer retinal layers corresponding to the placoid lesions and disruption of the retinal pigment epithelium, external limiting membrane, ellipsoid layer and interdigitation zone. **e**-**f** ICG-A showing numerous patchy areas of focal hypofluorescence at the posterior pole and anterior to the equator

A 60-year-old white female presented with acute loss of vision in both eyes. She had experienced fever over the preceding few days, along with malaise and myalgias. Previous ocular and medical history was unremarkable. At presentation, visual acuity (BCVA) was 20/200 in the right eye, 20/160 in the left eye. The slit-lamp examination revealed quiet anterior chambers. Intraocular pressure was normal in both eyes. At fundus examination, multiple, yellow-white placoid lesions were evident at the posterior pole of both eyes. Fluorescein Angiography (FA) showed characteristic early hypofluorescence and late hyperfluorescence of the lesions (Fig. 1a) and Autofluorescence (AF) revealed patchy hypoautofluorescence in these areas (Fig. 1b). Spectral-Domain Optical Coherence Tomography (OCT) scans across the lesions showed disruption of the RPE, external limiting membrane, ellipsoid layer and interdigitation zone; the placoid lesions appeared as focal deposits of hyperreflective material at the level of the outer retinal layers (Fig. 1c, d). On ICG-A, numerous round patchy areas of focal hypofluorescence were observed at the posterior pole and hypofluorescent satellite lesions could be seen close to the edge of the optic disc and near the vascular arcades (Fig. 1e). Both eyes exhibited similar features.

**Fig. 2:**
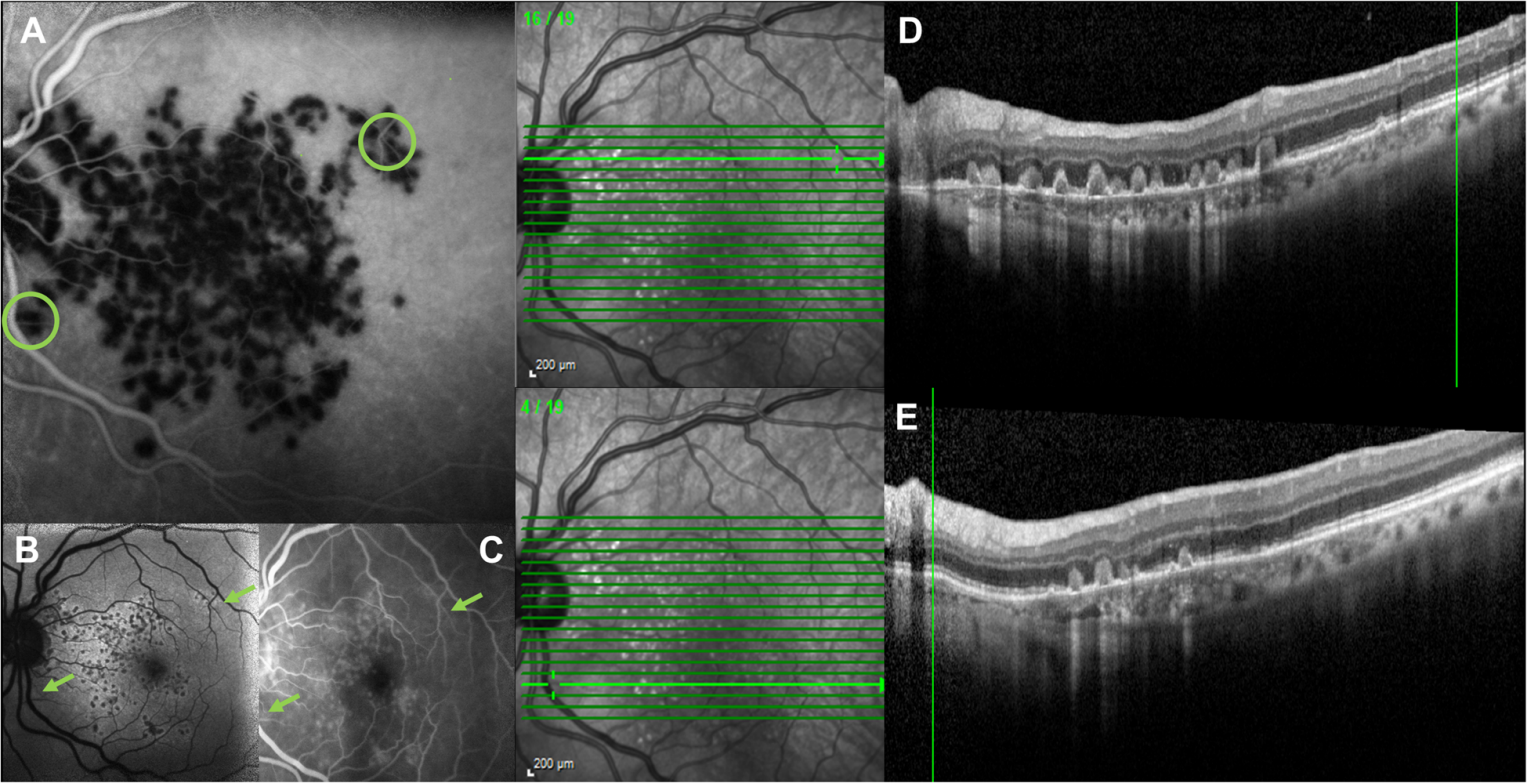
Imaging composite showing that the non-perfused areas may extend beyond the damage of the outer retina. Areas within the green circles on ICG-A image (**a**) are examples of choriocapillaris non-perfusions that are not associated with corresponding FA or AF abnormalities (green arrows indicate the corresponding locations in images **b** and **c**) nor with OCT subretinal deposits (the green vertical lines crossing the OCT images **d** and **e** represent positions corresponding to areas within the green circles)

Notably, all the focal lesions detected by FA, AF and OCT corresponded to areas of patchy hypofluorescence seen on ICG-A, whereas ICG-A also detected several additional hypofluorescent areas not associated with FA, AF or OCT abnormalities (Fig. 2).

As shown in Fig. 1, ICG-A also detected several placoid lesions anterior to the equator (Fig. 1f) that appeared much more diffuse and numerous, when compared with those visualized with FA.

**Fig. 3:**
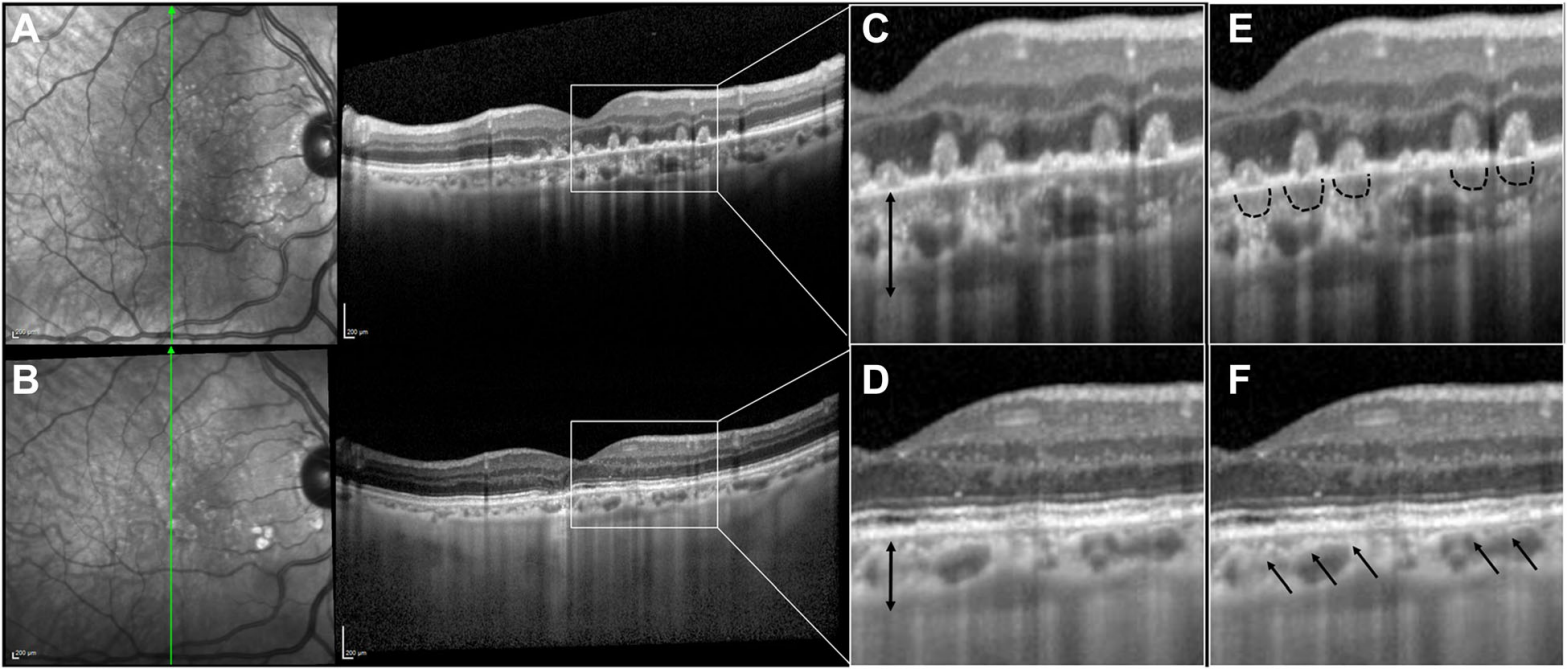
EDI-OCT findings in the acute and in the inactive phase of disease. **a** EDI-OCT in the acute phase of disease showing an increased full choroidal thickness when compared with the inactive phase (**b**). The magnified views highlight changes in the choroidal thickness (**c**-**d**). The same magnifications demonstrate focal areas of choriocapillaris thickening and hyporeflectivity beneath the placoid lesions (black dotted lines) during the active stage of the disease (**e**) and their regression in the acute phase of disease (**f**)

EDI-OCT was performed for quantitative and morphologic analysis of the choroid. The subfoveal choroidal thickness, measured vertically from the outer border of the RPE to the inner border of the sclera, was 238 μm (Fig. 3c). As previously reported in literature [7], focal areas of choriocapillaris thickening and hyporeflectivity were clearly visible on EDI-OCT beneath the placoid lesions during the active stage of the disease (Fig. 3e).

Besides a slightly raised erythrocyte sedimentation rate (ESR) and C-reactive protein (CRP) level, routine blood tests were normal. As were immunological exams and serology tests for infectious disease. Based on imaging and clinical features, the patient was diagnosed with APMPPE. Magnetic Resonance Imaging (MRI) of the brain and neurological examination excluded neurologic involvement. The patient was treated with prednisone 100 mg, which was tapered gradually over 2 weeks.

**Fig. 4:**
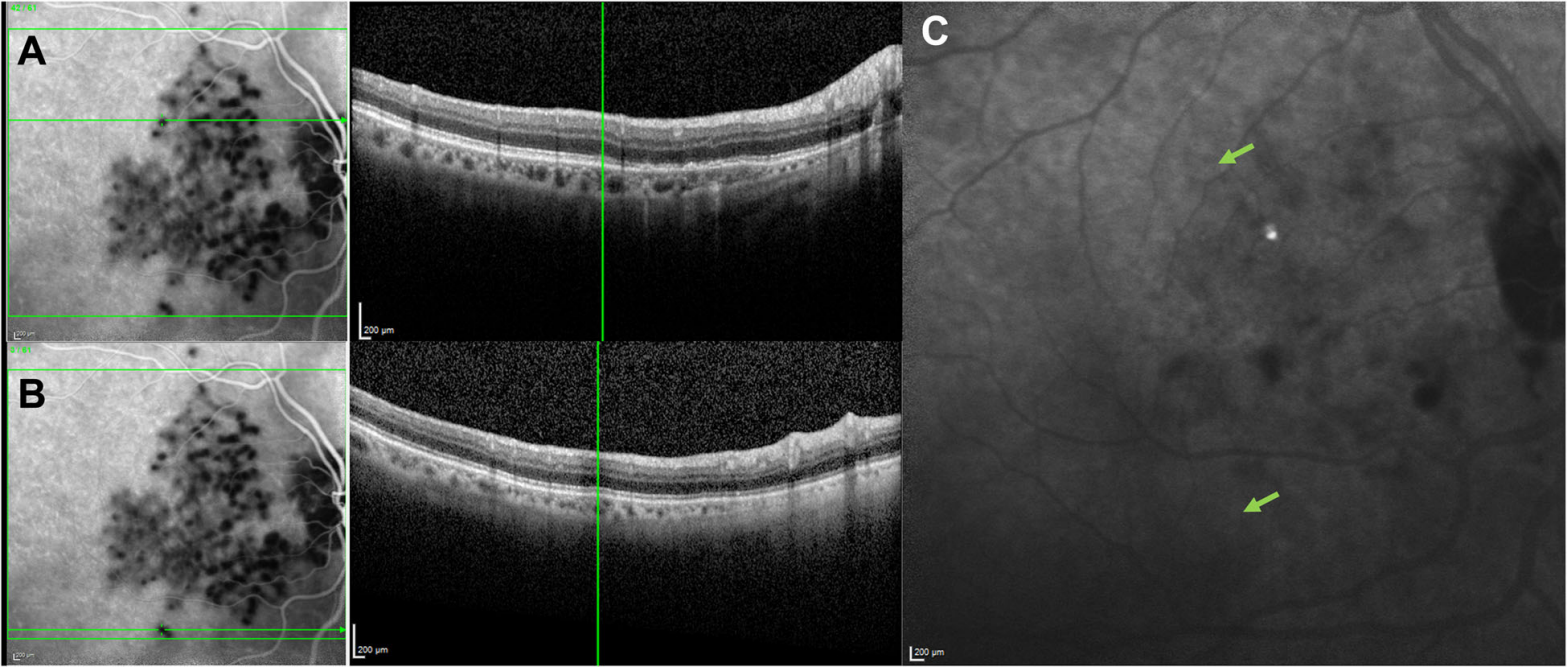
Regression of outer retinal abnormalities on OCT preceding the recovery of choroidal perfusion abnormalities on ICG-A. **a**-**b** Combined ICG-A and OCT during the ongoing process of remission. The restoration of outer retinal abnormalities is detectable with OCT, as shown in the right part of **a** and **b** images, while persistent patchy hypofluorescences can be identified in the corresponding locations with ICG-A, as shown in the left part of the images (the green orizontal lines crossing the ICG-A images highlight examples of persisting focal non-perfused areas, while the vertical lines crossing the OCT images represent the corresponding locations). **c** ICG-A in the inactive stage of disease showing the regression of patchy hypofluorescences; green arrows indicate the locations corresponding to previous focal hypoperfusion areas in **a** and **b** images

Notably, in follow-up examinations, the restoration of visual acuity and of outer retinal abnormalities, noted on SD-OCT from 10 days after the acute presentation, preceded the recovery of choroidal perfusion abnormalities detected with ICG-A. In fact, during the ongoing process of remission, while OCT scans showed the progressive regression of the hyperreflective placoid lesions and restoration of outer retinal abnormalities, ICG-A detected persisting patchy hypofluorescence in the corresponding locations (Fig. 4). This long-standing choroidal perfusion defect could not be detected by OCT.

Two months later, BCVA improved to 20/25 in both eyes and the patient was in good health. CPR level and ESR were normalized.

In the inactive stage of disease, EDI-OCT scans revealed a reduced full choroidal thickness compared with the acute phase of the disease (Fig. 3d). The subfoveal choroidal thickness was reduced to 156 μm. EDI-OCT also showed the regression the focal areas of choriocapillaris thickening and hyporeflectivity beneath the placoid lesions (Fig. 3f). On ICG-A, isofluorescence in the areas corresponding to previous placoid lesions could be detected as a sign of normal reperfusion of the choriocapillaris (Fig. 4c). Fundus examination showed areas of retinal pigment changes, but no active placoid lesions.

## Discussion

The exact etiology of APMPPE remains unknown. The disorder was initially presumed to primarily affect the RPE [1]. Conversely, subsequent evidence has suggested the choroid as the primary site of involvement [2–6, 8]. Moreover, de Vries et al. [9] proposed that APMPPE might be caused by choroidal granulomas and could be part of a generalized granulomatous disease, based on histopathological findings from a case of severe APMPPE showing granulomas just beneath the RPE.

As ICG-A and EDI-OCT enable excellent visualization of morphologic changes in the choroidal structure, they have been widely used as essential imaging techniques for diagnosis, management and understanding of the pathophysiology of many diseases involving the choroidal layer. Since the choroid is presumed to be the primary site of involvement in APMPPE, these diagnostic tools may be used to analyze the morphological changes occurring in the course of the disease, and to elucidate the pathologic mechanism behind the disorder.

Multimodal imaging findings in APMPPE have been previously reported, including the evaluation of features from ICG-A and EDI-OCT [5–7, 10, 11]. However, some controversial issues still remain. For example, whether the hypofluorescence observed on ICG-A results from choriocapillaris nonperfusion, from granulomas, or from blockage by the placoid lesions is debated. Moreover, although ICG-A and EDI-OCT evaluations of lesions in both active- and inactive stages of the disease have been reported [5, 7], no combined ICG-A and OCT analysis of the areas of hypoperfusion during the ongoing remission phase of the disease has so far been described, thus to elucidate the process of resolution of placoid lesions and to clarify at which site the definitive regression of disease is detectable.

Our findings show that ICG-A may allow the detection of several hypofluorescent areas that are not associated with FA, AF or OCT abnormalities. This finding excludes blockage by placoid lesions as the cause of the hypofluorescence observed with ICG-A. These hypofluorescent areas could be interpreted as sites at which choriocapillaris non-perfusion has not led to consequent alterations of the overlying retinal structures. Similarly, in some previously reported cases, the placoid lesions were reported to be less numerous than the choroidal abnormalities as determined with FA and ICG-A. Notably, in our report, a combined ICG-A and EDI-OCT analysis of the evolution of lesions during the ongoing remission process of disease was also performed, which compared the regression of abnormalities on both imaging techniques. As seen in Fig. 4a-b, the regression of the hyperreflective placoid lesions and restoration of outer retinal abnormalities on OCT, preceded the restoration of choroidal perfusion abnormalities in the corresponding locations on ICG-A, showing that the choroidal injury might be more persistent than RPE damage. This finding suggests that the choroidal layer, besides the primary site of involvement, is also the more severely- and longer-endured injured structure in this disorder. In our case, this long-standing choroidal perfusion defect could not be detected by OCT. In light of this finding, the definitive remission of the disease appears to be more accurately detectable with ICG-A than with OCT alone.

As seen in Fig. 4c, in the inactive stage, isofluorescence was detected on ICG-A, as a sign of definitive remission of the placoid lesions. This final isofluorescence demonstrated the previous persistent hypofluorescences as signs of activity of disease, rather than expression of inactive lesions healed with choriocapillary atrophy.

The EDI-OCT evaluation of choroidal thickness showed an increased thickness in the acute phase of the disease with a subsequent regression during the remission. This finding additionally supports the choroidal injury in APMPPE. In fact, if the damage were at the level of the RPE, one would expect to find no differences in the choroidal thickness in the acute phase of the disease. As previously reported [7], focal areas of choriocapillaris thickening and hyporeflectivity were detected on EDI-OCT beneath the placoid lesions during the active stage of the disease, as a sign of choriocapillaris malperfusion. Conversely, no homogenous lower/hyporeflective choroidal lesions resembling those seen in granulomatous disease [12] were detected with EDI-OCT examination.

## Conclusion

In conclusion, our report describes the ICG-A and EDI-OCT features in a case of APMPPE, with the following findings:

- Several ICG-A lesions that were not associated with corresponding FA, AF or OCT abnormalities.
- A long-standing choroidal perfusion defect that could not be detected by OCT alone.
- A transient choroidal thickening in the acute phase of the disease.
- No areas of choroidal abnormalities resembling granulomas.

These findings support choroidal hypoperfusion as the primary mechanism overlying the pathogenesis of the disease, and suggest that the non-perfused areas may extend beyond damage of the outer retina. Combined choroidal imaging with ICG-A and EDI-OCT provides detailed morphologic information of abnormalities in APMPPE and can accurately demonstrate definitive resolution of lesions.

## Abbreviations

AF: Fundus Autofluorescence
APMPPE: Acute Posterior Multifocal Placoid Pigment Epitheliopathy
BCVA: Best corrected visual acuity
CRP: C-reactive protein
EDI-OCT: Enhanced Depth Imaging Spectral-Domain Optical Coherence Tomography
ESR: Erythrocyte sedimentation rate
FA: Fluorescein angiography
ICG-A: Indocyanine Green Angiography
MRI: Magnetic Resonance Imaging
RPE: Retinal pigment epithelium
SD-OCT: Spectral-Domain Optical Coherence Tomography
